# Differential item functioning for items in Berger’s HIV Stigma Scale: an analysis of cohorts from the Indian, Swedish, and US contexts

**DOI:** 10.1007/s11136-018-1841-4

**Published:** 2018-03-24

**Authors:** Maria Reinius, Deepa Rao, Lisa E. Manhart, Maria Wiklander, Veronica Svedhem, John Pryor, Randall Mayer, Bambi Gaddist, Shuba Kumar, Rani Mohanraj, Lakshmanan Jeyaseelan, Lena Wettergren, Lars E. Eriksson

**Affiliations:** 1 Department of Learning, Informatics, Management and Ethics, Karolinska Institutet, SE-171 77 Solna, Sweden; 2 Department of Neurobiology, Care Sciences and Society, Karolinska Institutet, SE-141 83 Huddinge, Sweden; 3 Department of Infectious Diseases, Karolinska University Hospital, SE-141 86 Huddinge, Sweden; 4 School of Health Sciences, City, University of London, London, EC1V 0HB UK; 5 Department of Medicine, Huddinge, Unit of Infectious Diseases, Karolinska Institutet, 141 86 Huddinge, Sweden; 6 Department of Global Health and Department of Psychiatry and Behavioral Sciences, Harborview Medical Center, University of Washington, 325 9th Ave, UW Campus Mailbox Number 359931, Seattle, WA 98104 USA; 7 Department of Epidemiology, Harborview Medical Center, University of Washington, 325 9th Ave, UW Campus Mailbox Number 35993, Seattle, WA 98104 USA; 8 Department of Psychology, Illinois State University, Normal, IL USA; 9 Iowa Department of Public Health, 321 E. 12th St, Des Moines, IA 50319-0075 USA; 10 Joseph H. Neal Wellness Center Dba SC HIV Council, 1813 Laurel Street, Columbia, SC 29201 USA; 11 Department of Biostatistics, Christian Medical College, Vellore, India; 12 Department of Social Sciences, Samarth, Chennai, India; 13 Department of Women′s and Children′s Health, Karolinska Institutet, SE-171 77 Stockholm, Sweden

**Keywords:** HIV, Stigma, The HIV stigma scale, Differential item functioning, Item Response Theory, Psychometrics

## Abstract

**Purpose:**

To examine whether items in Berger’s HIV Stigma Scale function differently with persons of different age, gender, and cultural backgrounds.

**Methods:**

Secondary data from cohorts, collected in South India (*n* = 250), Sweden (*n* = 193), and the US (*n* = 603) were reanalyzed to evaluate DIF within, between, and across these cohorts. All participants had answered the revised version of the HIV stigma scale consisting of 32 items forming the subscales *Personalized stigma, Disclosure concerns, Concerns about public attitudes*, and *Negative self-image*. Differential Item Functioning (DIF) for these items was assessed using hybrid ordinal regression-IRT technique. When DIF was detected, the cumulative impact of DIF on individual subscale scores was evaluated.

**Results:**

DIF was detected for 9 items within, between, or across cohorts, but the DIF was negligible in general. Detected DIF between the Swedish and Indian cohorts had a cumulative salient impact on individual scores for the subscale *Disclosure Concerns; Disclosure concerns* were overestimated in the Swedish cohort and both over- and underestimated in the Indian cohort.

**Conclusions:**

The items in the 32-item version of the HIV stigma scale did not seem to be particularly prone to present DIF. The DIF between the Indian and Swedish cohort for items in the subscale *Disclosure Concerns* could, however, result in both type I and type II errors if scores should be compared between the Indian and Swedish cohort.

**Electronic supplementary material:**

The online version of this article (10.1007/s11136-018-1841-4) contains supplementary material, which is available to authorized users.

## Background

Since the beginning of the pandemic, people with HIV have been stigmatized. Mahajan [1] defines HIV-related stigma as when people living with HIV are labeled, stereotyped, experience separation and status loss, and become discriminated both on an individual and structural level. Across different contexts perceived HIV-related stigma is associated to poor mental and physical health for persons living with HIV [2–5]. Even in the current era of efficient treatment, making HIV a chronic illness with normal life expectancy where treatment is generally available, people living with HIV are exposed to and relate to HIV-related stigma [6, 7] and stigma has been found to be a common barrier to treatment and prevention [8, 9].

Stigma has sometimes been understood as an individual process, where stigma is constituted of what some individuals do to others [10], whereas other scholars have argued that an individual perspective of stigma only may be relevant in highly individualized countries (as the US or some parts of Europe) [11]. Parker and Aggleton [11] argue that stigma, where it appears, is strongly related to the specific context of culture and power, and that the social and cultural phenomenon of stigma may be linked to actions of whole groups of people. Some known aspects of HIV-related stigma are also clearly cultural specific, as they, for example, relate to specific religious beliefs [12].

The efforts to reduce HIV-related stigma have yet not matched the magnitude of the problem [13]. Parker and Aggleton suggest that the diversity and complexity of HIV-related stigma makes it difficult to grasp in a programmatically useful way [11]. Ogden and Nyblade, on the other hand, argue that differences in HIV-related stigma across cultures are largely superficial and that stigma is expressed remarkably consistent across contexts [13]. Valid and reliable instruments for measuring HIV-related stigma are essential for stigma research, for evaluation of stigma-reducing interventions, and for monitoring and understanding experiences of HIV-related stigma [10, 14, 15]. Based on findings that suggest consistencies in HIV-related stigma across cultures [13], it would be valuable to have measures of HIV-related stigma that are valid across cultures, thus enabling measurement of changing trends in HIV-related stigma over time and across contexts.

One of the many instruments designed to measure HIV-related stigma perceived by persons living with HIV is the HIV Stigma Scale by Berger et al. [16]. Although developed and originally found valid and reliable in a US context [16], the instrument has been translated and found to be relevant and valid across various countries and cultures [17, 18]. These findings suggest that HIV-related stigma as assessed by the Berger’s scale is, to some extent, universal. For example, the HIV Stigma Scale has been used to compare levels of perceived HIV-related stigma among persons living with HIV in Kenyan, Puerto Rican, and the United States contexts [3], where Hispanics reported significantly higher levels of stigma than persons from the African continent. When used in Kenya, Puerto Rico, and the United States, the HIV Stigma Scale showed good internal consistency measured by Cronbach’s α for the combined data. The authors, however, did not explore differential item functioning for items in the HIV Stigma Scale, and it is possible that participants from different countries interpreted the items differently, resulting in bias.

Participants’ interpretation of scales can be assessed with methods based on Item Response Theory (IRT) and analysis of Differential Item Functioning (DIF). As items in the HIV Stigma Scale are statements that participants are requested to agree or disagree with on a four-point Likert scale, the probability that an individual will agree with the statement on a certain item can, according to IRT, be seen as a mathematical function of how stigmatized the person is and how severe the stigma is that the item captures. Items in the HIV Stigma Scale would be considered to have DIF if participants with different sociodemographic backgrounds have unequal probabilities of agreeing with statements in the items, while experiencing the same level of stigma [19]. For example, an analysis of DIF in an American sample, showed that black, non-Hispanic persons and white, non-Hispanic persons with the same level of stigma, had different probabilities of agreeing with items in the HIV Stigma Scale, indicating that persons with different backgrounds may experience HIV-related stigma differently [20].

In addition to its use in several studies in the United States [21–23], the HIV Stigma Scale has been used, for example, in Sweden [18] and South India [17], providing an ideal opportunity to evaluate whether DIF occurs. The HIV Stigma Scale has been translated into the respective native languages, back translated into English, and checked for comparability with the original English questionnaire [17, 18]. The English, Tamil, and Swedish versions of the instrument could therefore be considered consistent regarding content and all items were found relevant for both a Swedish and a South Indian context by both experts and people living with HIV in each country [17, 18]. There are, however, results from the psychometric validation from both the Swedish and the Indian contexts that may indicate that at least parts of the concept of HIV-related stigma are culturally embedded. For the Swedish version, a high rate of missing responses were found for an item regarding the risk of losing employment if one’s HIV status is disclosed, where written comments in the margin of the questionnaire (“Does this happen in Sweden?”) indicated that some Swedish respondents found this item irrelevant [18]. In validation of the Indian version of the HIV Stigma Scale, respondents had difficulties understanding the four-point Likert scale and the word “unclean” in the item “Having HIV makes me feel unclean” was often misinterpreted to mean “personal hygiene” [17].

Although the HIV Stigma Scale has been used for measuring stigma in a wide range of different contexts, it is not clear whether items in the HIV Stigma Scale are interpreted differently by persons with different backgrounds. The aim of the present study was, therefore, to examine whether items in the HIV Stigma Scale function differently with regard to gender and cultural background.

## Method

### Overview

The present work builds on secondary analysis of data collected using the HIV Stigma Scale in India [17], Sweden [18], and three areas in the US, South Carolina [24], Iowa [24], and the city of Chicago [20]. Item-level data from these studies were used to evaluate items for DIF between, within, and across the cohorts. A hybrid ordinal logistic regression—IRT approach [25] was used for DIF detection, using the lordif-package [26] with R statistics [27]. When DIF was detected, we evaluated the cumulative impact that the detected DIF had on individual IRT scores [28].

### Data source

Data from the Swedish cohort were collected at the Karolinska University hospital in Stockholm in 2013 from 193 persons living with HIV [18]. Data for the South Indian cohort were collected from 2007 to 2008 through networks caring for men and women living with HIV in the state of Tamil Nadu. Participants were residing in and around the cities Chennai (*n* = 150), which is an urban area and Vellore (*n* = 100), a semirural area [17]. Data from the United States were collected at two different time points in three different states. Between 2005 and 2010, data were collected through Ryan White II/III clinics and a community-based organization providing medical case management in South Carolina (*n* = 210) [20], from Iowa Department of Public Health Title II case-managed clients in Iowa (*n* = 331) [20], and a private University Hospital HIV Clinic in Chicago (*n* = 62) [24].

Available data on sociodemographic information differed across location. Information about gender and age was available for all participants from Sweden, India, and Chicago (US). Gender was categorized as male, female, or transgender in data collected in Chicago, with one participant categorized as transgender. For all other data, gender was categorized as male or female. Differences between cohorts were assessed with χ^2^-test for gender and ANOVA with Tukey post hoc analysis for age.

### The HIV Stigma Scale

The HIV Stigma Scale is designed to measure four dimensions of perceived HIV-related stigma among persons living with HIV: *Personalized stigma, Disclosure concerns, Concerns about public attitudes*, and *Negative self-image. Personalized stigma* is proposed to capture perceived consequences of other people knowing about one’s HIV infection. *Disclosure concerns* captures concerns that one can have over disclosing one’s HIV status to others. *Concerns about public attitudes* includes concerns that one can have over what other people think about people with HIV. *Negative self-image* includes negative feelings that one can have about oneself related to one’s HIV, (e.g., feeling unclean or not as good as others because of HIV) [16]. In scale construction of the original HIV Stigma Scale, items were assigned to dimensions following an exploratory factor analysis where a majority of items cross-loaded. Seventeen items were therefore assigned to more than one dimension. Subscales were subsequently refined to consist of unique items in a revised 32-item version [29]. Since DIF analyses with the lordif-package assume unidimensionality in the latent trait, where all items measure a single concept [19], the items that compose the revised 32-item version of the HIV Stigma Scale were used in the present work. The instrument includes 32 statements related to HIV stigma that are completed on a four-point Likert scale, ranging from 1 (“completely disagree”) to 4 (“completely agree”), and item scores for each subscale are summed into subscale scores [16]. Higher scores indicate higher levels of perceived stigma for all four subscales.

### Unidimensionality

Unidimensionality for scales was assessed through confirmatory factor analysis (CFA), where all items in each scale were specified to load onto one single factor, respectively. Since responses to the HIV stigma scale are ordinal, the CFA models were fitted using weighted least squares means and variance (WLSMV) [30]. Measures of model fit used were as follows: Chi-square (expected to be non-significant if the model showed acceptable fit to the data), the Comparative Fit Index and the Tucker–Lewis Index (CFI and TLI, cut-off > 0.95), and the root mean square error of approximation (RMSEA, cut-off < 0.08) [31, 32]. Since Chi-squares were significant in all CFA’s but three and RMSEA exceeded the cut-off for two subscales regarding the Swedish cohort, we also assessed the dimensionality of the models exploratory with parallel analysis and the Empirical Kaiser Criterion [33].

### DIF detection

A combined ordinal logistic regression—IRT approach for DIF detection and analysis of DIF impact [25]—was employed, as implemented in the lordif-package [26]. First, IRT item parameter estimates were obtained, using the mirt package [34], according to the graded response model [35]. The graded response model was chosen over the partial credit model since all items had the same number of response categories (four). Missing values were not imputed; participants were excluded listwise in each separate analysis if missing values were present. The minimum cell count for not collapsing response categories was set to five. Three ordinal regression models were examined for each item:

$${\text{Model 1 }}\left( {{\text{no DIF}}} \right):logitP({u_{\text{i}}} \ge k)={\alpha _{\text{k}}}+{\beta _1} \times \theta$$

$${\text{Model 2 }}\left( {{\text{uniform DIF}}} \right):logitP({u_{\text{i}}} \ge k)={\alpha _{\text{k}}}+{\beta _1} \times \theta +{\beta _2} \times group$$

$${\text{Model 3 }}\left( {{\text{non}} - {\text{uniform}}} \right){\text{ DIF}}:logitP({u_{\text{i}}} \ge k)={\alpha _{\text{k}}}+{\beta _1} \times \theta +{\beta _2} \times group+{\beta _2} \times \theta \times group,$$

where *P(u*_i_ ≥ *k)* represented probabilities that item response *u*_i_ is *k* or higher [26], *θ* was the latent trait in the present work, four dimensions of perceived HIV-related stigma *(Personalized stigma, Disclosure Concerns, Concerns of public attitudes* and *Negative Self-image)*. Group was a variable indicating group (country or gender) and (*θ* × group) represented the interaction between the latent trait and group. Pseudo *R*^2^ changes (Nagelkerke) were used as a criterion for DIF detection. When pseudo *R*^2^ changes for an item exceeded the empirical threshold, that item was flagged for DIF. Pseudo R^2^ changes between Model 1 and Model 2 indicate a uniform DIF, with a constant effect for different levels of stigma while pseudo *R*^2^ changes between Model 2 and 3 indicate a non-uniform DIF, with an effect that varied conditional on levels of stigma. The procedure was extended for multiple groups in analysis of DIF between cohorts [26]. Empirical thresholds for DIF detection were generated through Monte Carlo simulations of DIF-free samples [26]. The empirical thresholds were set to be above the highest value identified in the Monte Carlo simulation, identical for all items in each separate analysis, but varying between analyses [26]. The Monte Carlo-simulated item-level thresholds are presented in Online Resource 1.

An iterative process was used to prevent false-positive and false-negative results that may occur when many items with DIF are included in analysis [25]. Trait estimates were updated using the items that were not flagged for DIF and used as a new threshold for DIF detection. The logistic regression was then repeated and if different items were flagged for DIF than in the initial calculation, trait estimates were updated again based on the DIF-free samples in the most previous round. This procedure was repeated until the same items were flagged for DIF in two successive rounds [26].

Based on the knowledge that HIV-related stigma can be experienced differently by persons living with HIV from different backgrounds [20], a series of DIF analyses was performed between and within each cohort, comparing groups with different backgrounds. For the US cohort, DIF was examined between Black, non-Hispanic persons and White, non-Hispanic persons (data collected in Chicago excluded), as earlier examined by Rao et al. [20]. The US cohort was then combined with the Swedish and South Indian cohort and DIF was assessed between persons living in Sweden, the US, and South India. Since respondents in the different cohorts differed with respect to gender, we also examined whether the HIV Stigma Scale had DIF between men and women. Transgender was in analysis coded as missing since this category only included one respondent. DIF related to gender was assessed for the three cohorts combined and are referred to as DIF across cohorts.

### Magnitude of detected DIF

There are no minimal clinically important score differences specified for the HIV Stigma Scale to define a salient DIF. We therefore evaluated the individual-level cumulative impact of detected DIF through comparing unadjusted IRT scores (with DIF) with adjusted IRT scores (accounted for DIF). Unadjusted IRT scores were subtracted from adjusted IRT scores and if individual IRT scores were unaffected by DIF, this difference would be zero [28]. Differences that were equal to or exceeded a small effect size (i.e., 0.20) were considered to be salient [36].

## Results

### Demographic characteristics

Demographic characteristics by cohort are shown in Table 1. The US sample had a significantly higher proportion of males compared to the Indian sample (66% vs. 50%, χ^2^ = 60.8, *p* < 0.001). Further, the one-way ANOVA showed a statistical significance between group effect of age (F(2,495) = 126.7, *p* < 0.001) (data on participants’ age were not available for Iowa and South Carolina). Post hoc comparisons using the Tukey HSD test indicated that the mean age of Indian respondents (mean = 35, SD = ± 6) was significantly lower than the mean age of respondents from the US (mean = 43, SD = ± 11) and Sweden (mean = 49, SD = ± 12), *p* < 0.001. Mean age for respondents from the US (mean = 43, SD = ± 11) was also significantly lower than the mean age of Swedish respondents (mean = 49, SD = ± 12), *p* < 0.001.

**Table 1:** Demographic characteristics of respondents to the HIV Stigma Scale in South India [11], Sweden [12], and the US [11, 14, 18]

Cohort	*n*	Gender %Female	Age^b^ Mean, range (SD)
South India	250	50^a^	35, 18–49 (6)^c,d^
Sweden	188	43	49, 19–83 (12)^c,e^
The United States	598	34^a^	43, 23–68 (11)^d,e,f^
All	1036	40	41, 18–83 (11)

Respondents with missing values in one or more of the HIV stigma subscales were excluded

^a^Significant difference between India and the United States (χ^2^ = 60.8, *p* < 0.001)

^b^Overall significant difference (ANOVA): F(2,495) = 126.7, *p* < 0.001

^c^Significant difference between India and Sweden, Tukey post hoc test, *p* < 0.001

^d^Significant difference between India and Chicago, Tukey post hoc test, *p* < 0.001

^e^Significant difference between Chicago and Sweden, Tukey post hoc test, *p* < 0.001

^f^Age only available for respondents from Chicago

### Unidimensionality

The unidimensional models yielded reasonable fit for all data combined and for the South Indian and US cohort separately (Table 2) which suggested suitability for unidimensional IRT analysis. For the Swedish cohort, unidimensionality was not supported in the confirmatory analysis for the scales *Disclosure concerns* and *Concerns with public attitudes*. The exploratory analysis using parallel analysis suggested one-factor solution in all analyses. The Empirical Kaiser Criterion, however, suggested two-factor solutions for *Disclosure concerns* and *Negative self-image* regarding the South Indian cohort and for *Disclosure concerns* regarding the Swedish cohort.

**Table 2:** Assessment of unidimensionality for all subscales in the 32-item HIV Stigma Scale

Subscale	Fit measures from CFA^a^			
	All data combined	Indian cohort	Swedish cohort	US cohort
Personalized stigma				
*n*	960	244	157	559
Chi-square(df = 44)	148.37*	75.0544*	67.12*	150.40*
CFI	0.998	0.995	0.999	0.998
TLI	0.998	0.993	0.999	0.997
RMSEA	0.050	0.054	0.058	0.066
Disclosure concerns				
*n*	992	250	168	574
Chi-square(df = 20)	134.66*	24.74, ns	51.87*	90.50*
CFI	0.991	0.996	0.992	0.992
TLI	0.997	0.994	0.989	0.989
RMSEA	0.076	0.031	0.098	0.078
Concerns about public attitudes				
*n*	965	246	159	560
Chi-square(df = 9)	39.48*	4.38, ns	38.38*	34.63*
CFI	0.997	1.000	0.987	0.997
TLI	0.995	1.008	0.979	0.995
RMSEA	0.059	< 0.001	0.114	0.071
Negative self-image				
*n*	1010	248	180	582
Chi-square(df = 14)	45.65*	35.79*	9.72, ns	38.28*
CFI	0.997	0.981	1.000	0.996
TLI	0.995	0.972	1.003	0.994
RMSEA	0.047	0.079	< 0.001	0.055

	Number of factors proposed to retain by EKC^b^			
Personalized stigma	1	1	1	1
Disclosure concerns	1	**2**	**2**	1
Concerns about public attitudes	1	1	1	1
Negative self-image	1	**2**	1	1

Figures in bold indicate that the subscale was not unidimensional according to EKC

*p < 0.001

^a^Confirmatory factor analysis of unidimensional models, fitted using the weighted least squares mean and variance (WLSMV)

^b^The Empirical Kaiser Criterion

### Fit of the graded response models

The lowest number of endorsements for a category in a group was six. Since the minimum cell count was set to five, none of the response categories were collapsed.

### DIF detection

Nine items in the HIV Stigma Scale were flagged for DIF with pseudo *R*^2^ change exceeding the thresholds generated through Monte Carlo simulations of DIF-free samples (Table 3). Both uniform (with a constant effect for different levels of stigma) and non-uniform DIF findings (with an effect that varies depending on levels of stigma) were detected.

**Table 3:** Differential item functioning findings for items in Berger’s HIV Stigma Scale (32-item version) within, between, and across the three cohorts

Items	DIF within cohorts U.S. cohort^a^			DIF between cohorts^b^			DIF across cohorts DIF related to gender^c^		
	Uniform DIF		Non-uniform DIF	Uniform DIF		Non-uniform DIF	Uniform DIF		Non-uniform DIF
Personalized stigma^d^									
Threshold^h^		0.01			0.01			0.01	
18. Some people who know I have HIV have grown more distant	0.0002		0.0002	0.0007		0.0029	0.0031		< 0.0001
24. I have been hurt by how people reacted to learning I have HIV	0.0003		0.0004	0.0061		0.0005	0.0079		< 0.0001
26. I regret having told some people that I have HIV	0.0016		0.0022	0.0046		0.0003	0.0003		< 0.0001
28. Some people avoid touching me once they know I have HIV	0.0003		0.0000	0.0077		0.0007	0.0001		< 0.0001
29. People I care about stopped calling after learning I have HIV	0.0012		0.0011	0.0016		0.0043	0.0002		< 0.0001
32. People don’t want me around their children once they know I have HIV	0.0001		0.0000	0.0035		0.0005	0.0001		< 0.0001
33. People have physically backed away from me when they learn I have HIV	0.0000		0.0009	0.0008		0.0018	0.0000		< 0.0001
35. I have stopped socializing with some people because of their reactions to my having HIV	0.0005		0.0002	0.0002		0.0000	0.0000		< 0.0001
36. I have lost friends by telling them I have HIV	0.0018		0.0014	0.0004		0.0016	0.0012		< 0.0001
38. People who know I have HIV tend to ignore my good points	0.0015		0.0004	*0.0115* ^h^		0.0038	0.0000		< 0.0001
39. People seem afraid of me once they learn I have HIV	0.0009		0.0014	0.0004		0.0002	0.0000		< 0.0001
Disclosure concerns^e^									
Threshold^h^		0.02			0.01			0.01	
1. In many areas of my life, no one knows that I have HIV	0.0048		0.0007	**0.0293** ^i^		**0.0204** ^i^	*0.0138* ^h^		0.0001
4. Telling someone I have HIV is risky	0.0015		0.0000	0.0005		0.0004	0.0000		0.0011
6. I work hard to keep my HIV a secret	0.0127		0.0010	**0.0517** ^i^		0.0061	0.0011		0.0002
17. I am very careful who I tell that I have HIV	0.0000		0.0093	**0.0219** ^i^		**0.0181** ^i^	0.0020		0.0000
21. I never feel the need to hide the fact that I have HIV (R)	0.0090		0.0049	**0.0242** ^i^		0.0009	0.0001		0.0025
22. I worry that people may judge me when they learn I have HIV	0.0057		0.0013	0.0023		0.0083	0.0002		0.0000
25. I worry that people who know I have HIV will tell others	0.0000		0.0039	0.0015		0.0012	0.0003		0.0013
37. I have told people close to me to keep the fact that I have HIV a secret	0.0014		0.0004	0.0025		**0.0191** ^i^	0.0010		0.0011
Concerns about public attitudes^f^									
Threshold^h^		0.01			0.01			0.01	
5. People with HIV lose their jobs when their employers find out	0.0090		0.0006	*0.0188* ^h^		0.0033	< 0.0001		0.0015
9. People with HIV are treated like outcasts	0.0024		0.0019	0.0070		0.0066	< 0.0001		0.0003
10. Most people believe that a person who has HIV is dirty	0.0001		0.0014	0.0019		0.0004	< 0.0001		0.0004
14. Most people think that a person with HIV is disgusting	0.0004		0.0005	0.0061		0.0006	< 0.0001		0.0000
16. Most people with HIV are rejected when others find out	0.0034		0.0001	0.0017		0.0038	< 0.0001		0.0001
20. Most people are uncomfortable around someone with HIV	0.0000		0.0048	0.0008		0.0034	< 0.0001		0.0000
Negative self-image^g^									
Threshold^h^		0.02			0.01			0.01	
2. I feel guilty because I have HIV	0.0032		0.0002	0.0006		0.0048	0.0003		0.0000
3. People’s attitudes about HIV make me feel worse about myself	0.0005		0.0000	*0.0174* ^h^		0.0010	0.0021		0.0006
7. I feel I am not as good a person as others because I have HIV	0.0000		0.0000	0.0026		0.0005	0.0001		0.0009
8. I never feel ashamed of having HIV (R)	0.0001		0.0064	*0.0502* ^h^		0.0056	0.0002		0.0037
12. Having HIV makes me feel unclean	0.0015		0.0002	0.0044		0.0004	0.0003		0.0000
15. Having HIV makes me feel like I’m a bad person	0.0013		0.0004	0.0044		0.0007	0.0001		0.0008
23. Having HIV in my body is disgusting to me	0.0004		0.0003	0.0045		0.0032	0.0021		0.0010

Empirical thresholds for DIF detection were generated through Monte Carlo simulations of DIF-free samples

^a^DIF between White non-Hispanics and Black non-Hispanics (data collected in Chicago excluded)

^b^DIF between persons living in Sweden, US, and South India

^c^DIF between men and women (transgender coded as missing)

^d^290 white non-hispanic and 207 Black non-Hispanic participants in within cohort analysis; 559 US, 244 South Indian, and 157 Swedish participants in between cohort analysis; 586 male and 371 female participants in analysis across cohorts

^e^303 White non-Hispanic and 209 Black non-Hispanic participants in within cohort analysis; 574 US, 250 South Indian, and 168 Swedish participants in between cohort analysis; 599 male and 390 female participants in analysis across cohorts

^f^296 White non-Hispanic and 206 Black non-Hispanic participants in within cohort analysis; 560 US, 246 South Indian, and 159 Swedish participants in between cohort analysis; 583 male and 379 female participants in analysis across cohorts

^g^308 White non-Hispanic and 212 Black non-Hispanic participants in within cohort analysis; 582 US, 248 South Indian, and 180 Swedish participants in between cohort analysis; 606 male and 401 female participants in analysis across cohorts

^h^Italic values indicate pseudo *R*^2^ change for items that were flagged for DIF, but where DIF did not have a salient impact on individual scores. The threshold for DIF detection was generated through Monte Carlo simulations of DIF-free items

^i^Bold values indicate items that were flagged for DIF and where DIF had a cumulative salient impact on individual IRT scores

#### DIF within cohorts—the US cohort

For the US cohort, DIF was examined between Black, non-Hispanic persons and White, non-Hispanic persons. No items were flagged for DIF within the US cohort.

#### DIF between cohorts

Nine items in the HIV Stigma Scale were flagged for either uniform, non-uniform, or both uniform and non-uniform DIF between persons living in Sweden, the US, and South India, respectively. One item for *Personalized stigma*, five items for *Disclosure concerns*, one item for *Concerns about public attitudes*, and two items for *Negative self-image* (Table 3).

#### DIF across cohorts—gender

One item was found to have non-uniform DIF between men and women (Table 3) (1. In many areas of my life, no one knows that I have HIV, belonging to the subscale *Disclosure concerns*).

### Magnitude of detected DIF

**Fig. 1:**
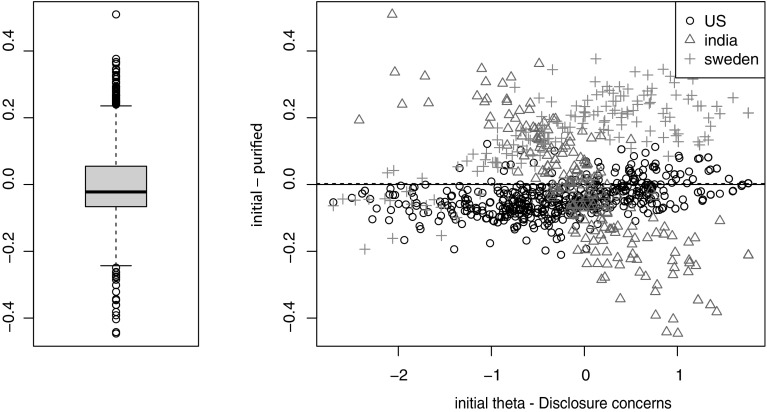
Cumulative individual-level DIF impact for subscale *Disclosure concerns* for DIF between persons living in South India (triangles), Sweden (plus signs), and the US (circles). The boxplot to the left shows differences in IRT score between using scores that ignore DIF and scores that account for DIF. In the scatterplot to the right, the difference scores (initial-purified) are plotted against the initial scores ignoring DIF (initial theta)

The detected DIF was, in general, negligible, since the cumulative impact of DIF on individual IRT scores was below 0.2. Salient individual-level impacts of DIF (exceeding 0.2) were, however, detected between cohorts for the subscales *Disclosure concerns*, which are detailed below and shown in Fig. 1.

#### DIF between cohorts—cumulative DIF impact on individual scores

Regarding DIF between cohorts, changes in individual IRT scores exceeded 0.2 and were therefore thought to be salient for the subscale *Disclosure concerns* (Fig. 1). Scores for respondents from the US were mainly unaffected by DIF, but DIF had a salient impact on scores for Indian and Swedish respondents, with effects in opposite directions. For *Disclosure concerns*, the HIV Stigma Scale seemed to overestimate scores for Swedish participants and both overestimate (for persons with low levels of *Disclosure concerns*) and underestimate (for persons with high levels of *Disclosure concerns*) scores for Indian participants (Fig. 1). The mean of the differences was close to zero, indicating that this DIF would not affect the mean score if levels of *Disclosure concerns* would be presented for persons from the Swedish, US, and South Indian cohort together.

## Discussion

The HIV Stigma Scale was developed for quantitative measurement of HIV-related stigma, as perceived by persons living with HIV. Though adapted and used in diverse contexts, this is the first time that the instrument has been assessed for gender-related DIF and DIF across different cultural contexts. The results of the present study indicate that the items in the HIV stigma scale were not especially prone to present culture-related DIF for the subscales *Personalized stigma, Concerns about public attitudes,* and *Negative self-image*. These subscales seem to cover aspects of stigma that in general are equally interpreted regarding content across the different groups investigated. These results indicate that levels of *Personalized stigma, Concerns about public attitudes,* and *Negative self-image* may be compared between the cohorts without the risk of results being culturally biased. Salient DIF was, however, found between the South Indian and Swedish cohorts for the subscale *Disclosure concerns*. The subscale *Disclosure concerns* measures concerns that one can have over disclosing one’s HIV status to others. The items that present DIF mainly covers the aspect that one’s HIV is a secret and we can only speculate about potential reasons for this DIF. Since India is more densely populated than Sweden, it may be likely that participants in the South Indian cohort live physically closer to family and neighbors than the participants in the Swedish cohort, making it harder for the South Indian participants to keep their HIV a secret. India has been characterized as a ‘collectivist’ society [37] and Sweden more of an individualistic society [38], particularly around interpersonal issues. According to Chadda and Deb [37] family is far more involved in the care of its members in the Indian society, compared to western societies. This might also make it more difficult for the South Indian participants to keep their HIV a secret from their family, compared to the Swedish participants. If keeping one’s HIV a secret is difficult, on the edge of being impossible, in the South Indian context, items like “I work hard to keep my HIV a secret” might have been perceived as irrelevant for South Indian participant, thus generating the detected DIF.

When Rao et al. [20] examined DIF between Black, non-Hispanic persons and White, non-Hispanic persons, nine items of the scale demonstrated DIF. Our present results did not replicate these findings, as no items were flagged for DIF between Black, non-Hispanic persons and White, non-Hispanic persons. A possible explanation for the differences in results could be that Rao et al. [20] examined the 40-item version of the HIV Stigma Scale, while the 32-item version was examined in the present study. Seven of the items, from the 40-item full version of the instrument, that demonstrated DIF in the earlier study by Rao et al. were items that cross-loaded in an exploratory factor analysis and therefore were excluded from the 32-item version of the instrument [29].

A common reason for DIF is a lack of translation equivalence [19]. The item “Having HIV makes me feel unclean” (Item 12), for example, could not be adequately translated to Tamil, since no words in Tamil captured the intended meaning of “unclean” [17]. This was adjusted for by letting the questionnaire be administered by professional raters in the South Indian cohort, who assured that item content was understood as intended [17]. A similar procedure was used for the Swedish cohort where members of the research team also were present to answer questions and assist respondents with explanations of items if needed [18]. The item “Having HIV makes me feel unclean” (Item 12) did not demonstrate DIF in the present analysis, but if the HIV Stigma Scale is used as a self-administered instrument as originally intended, the detected DIF may possibly be even more pronounced.

Unidimensionality was assessed with both confirmatory and exploratory methods. Since confirmatory methods indicated that some subscales might not be unidimensional, dimensionality was examined further with both parallel analysis and the Empirical Kaiser Criterion. Parallel analysis [39] supported unidimensionality for all subscales across all cohorts, while the Empirical Kaiser Criterion suggested two-factor solutions for the South Indian and Swedish cohort. Parallel analysis is an often recommended approach for dimensionality assessment [39]. The HIV stigma scale is, however, an instrument constituted of oblique, highly correlated factors [16, 18], and for this specific case the Empirical Kaiser Criterion has been shown to outperform parallel analysis [33]. We therefor conclude that the subscales *Disclosure concerns* and *Negative self-image* may be, at least, bi-factorial scales when used in the Swedish or South Indian context. These findings may have implications for the interpretation of the detected DIF, as multidimensionality can be mistaken for DIF [40].

There are several techniques available for DIF detection, and there is a lack of consensus regarding thresholds for detection of DIF [28]. As we used a hybrid ordinal logistic regression—IRT approach, other techniques may have produced different results. Since statistical significance not necessarily implies practical significance, we used a measure of effect size (changes in pseudo *R*^2^) as a criterion for when DIF should be detected [19]. Different sets of rules have been presented for when pseudo *R*^2^ should be considered to represent DIF [19], where Zumbo [41] suggested that cut-offs indicating moderate and large DIF should be 0.13 and 0.26, respectively, while the Jodoin and Gierl approach suggests cut-offs of 0.035 and 0.070 [42]. A cut-off level of 0.02 has also been commonly used and these different cut-offs can, unsurprisingly, produce very different numbers of items flagged for DIF [19]. In the present work we used Monte Carlo simulations, as implemented in the lordif-package [26], to generate empirical cut-offs. This rendered cut-offs as low as 0.01 for some analyses, which also resulted in detection of DIF that did not have a salient impact on individual scores. This low threshold for DIF detection was, however, set knowing that cultural DIF probably was adjusted for already in data collection and we sought to find patterns in present DIF that perhaps would have been more pronounced if questionnaires had been exclusively self-administered.

Limitations in the present work are that data were collected at different time points with over 5 years apart and that data from the United States and India were not collected with an intention to be representative for people living with HIV in the respective countries [17, 20, 24]. In those settings, the study populations represented a sub-set of all people living with HIV. The results, therefore, cannot be generalized to represent differences between countries. Furthermore, we do not know if the results would be replicated if data were collected at the same locations today. A recommended minimum sample size for ordinal logistic regression is 200 participants per group [19]. Thus, a limitation in the present study is the Swedish sample size of 157–180 participants depending on subscale analyzed. In a simulation study, sample sizes as low as 100 per group were sufficient to detect large DIF but not moderate DIF [43]. It is therefore possible that the present study failed to detect moderate but practically important DIF. Further limitations were that the datasets differed regarding gender and age, and we did not have access to other sociodemographic data. It is generally known that HIV-related stigma is linked to stigma related to other attributes, which can potentiate the power of stigmatization [1]; groups of people who are already exposed of racism, homophobia, sexism, or poverty are predisposed to greater HIV-related stigma [1]. As we did not have access to data other than ratings to the HIV stigma scale and gender and, to some extent, age, we were not able to control for potentially confounding variables that may have been a true source of DIF. For the HIV Stigma Scale it would also be interesting to analyze if DIF occurs between groups of different health statuses, e.g., persons who are virally suppressed or not, persons who have been living with HIV for a longer or shorter period of time.

Aside from these limitations, we propose that the results in the present work indicate that the items in the subscales *Personalized stigma, Concerns about public attitudes,* and *Negative self-image* in the HIV Stigma Scale are not especially prone to present salient DIF. However, the detected DIF between the Indian and Swedish cohort for the subscale *Disclosure concerns* did have a cumulative, albeit small, salient effect on individual IRT scores, which could result in both type I and type II errors if levels of *Disclosure concerns* should be compared between the Swedish and Indian cohorts. The detected DIF in the subscale Disclosure concerns could be a “true” differential item functioning, i.e., that persons from the Swedish and South Indian cohort respond differently to items regarding “keeping one’s HIV a secret” and “hiding one’s HIV,” respectively, even after accounting for their overall level of disclosure concerns. One possible explanation for this could be differences in the actual possibility to keep private things a secret in the Indian society, compared to the Swedish. An additional possible explanation could be that the unidimensional nature that the subscale *Disclosure concerns* seems to have in the Swedish and South Indian cohort may have caused a DIF due to multidimensionality. Apart from the cause of the detected DIF, we cannot recommend the subscales *Disclosure concerns* for comparisons between Sweden and South India.

The results in the present study, however, support the use of the subscales *Personalized stigma, Concerns about public attitudes,* and *Negative self-image* for comparisons of levels of stigma between the cohorts investigated. As the HIV Stigma Scale is being used to assess stigma in a wide range of different countries, we encourage researchers using the HIV Stigma Scale to cooperate across country borders and examine the cross-cultural validity of the instrument further. This would broaden the understanding of the extent and forms of stigma faced by people living with HIV in different countries.

## Electronic supplementary material

Below is the link to the electronic supplementary material.

Supplementary material 1 Item-level Monte Carlo thresholds for changes in pseudo R^2^ (100 replications) regarding DIF within cohorts. The data points are connected with lines to show a fluctuation across items, not implying a series. Numbers on the x-axis correspond to the order that items appear in the subscale, not the number that the item have in the instrument. These graphs show thresholds for three different pseudo R2 measures. The measure used in the article was Nagelkerke (EPS 2386 KB)

Supplementary material 2 Item-level Monte Carlo thresholds for changes in pseudo R^2^ (100 replications) regarding DIF between cohorts. The data points are connected with lines to show a fluctuation across items, not implying a series. Numbers on the x-axis correspond to the order that items appear in the subscale, not the number that the item have in the instrument. These graphs show thresholds for three different pseudo R2 measures. The measure used in the article was Nagelkerke (EPS 2401 KB)

Supplementary material 3 Item-level Monte Carlo thresholds for changes in pseudo R^2^ (100 replications) regarding DIF across cohorts. The data points are connected with lines to show a fluctuation across items, not implying a series. Numbers on the x-axis correspond to the order that items appear in the subscale, not the number that the item have in the instrument. These graphs show thresholds for three different pseudo R2 measures. The measure used in the article was Nagelkerke (EPS 2409 KB)
